# The Differential Effects of Leukocyte-Containing and Pure Platelet-Rich Plasma on Nucleus Pulposus-Derived Mesenchymal Stem Cells: Implications for the Clinical Treatment of Intervertebral Disc Degeneration

**DOI:** 10.1155/2018/7162084

**Published:** 2018-10-23

**Authors:** Jun Jia, Shan-zheng Wang, Liang-yu Ma, Jia-bin Yu, Yu-dong Guo, Chen Wang

**Affiliations:** ^1^School of Medicine, Southeast University, 87 Ding Jia Qiao Road, Nanjing, Jiangsu 210009, China; ^2^Department of Orthopaedics, Zhongda Hospital, School of Medicine, Southeast University, 87 Ding Jia Qiao Road, Nanjing, Jiangsu 210009, China; ^3^The First Clinical Medical School, Nanjing Medical University, 300 Guangzhou Road, Nanjing, Jiangsu 210029, China

## Abstract

**Background:**

Platelet-rich plasma (PRP) is a promising strategy for intervertebral disc degeneration. However, the potential harmful effects of leukocytes in PRP on nucleus pulposus-derived mesenchymal stem cells (NPMSCs) have seldom been studied. This study aimed at comparatively evaluating effects of pure platelet-rich plasma (P-PRP) and leukocyte-containing platelet-rich plasma (L-PRP) on rabbit NPMSCs in vitro.

**Methods:**

NPMSCs isolated from rabbit NP tissues were treated with L-PRP or P-PRP in vitro, and then cell proliferation and expression of stem cell markers, proinflammatory cytokines (TNF-*α*, IL-1*β*), production of ECM (extracellular matrix-related protein), and NF-*κ*B p65 protein were validated by CCK-8 assay, real-time polymerase chain reaction, enzyme-linked immunosorbent assay, immunofluorescence, and western blot respectively.

**Results:**

NPMSCs differentiate into nucleus pulposus-like cells after treatment of PRPs (P-PRP and L-PRP), and NPMSCs exhibited maximum proliferation at a 10% PRP dose. L-PRP had observably higher concentration of leukocytes, TNF-*α*, and IL-1*β* than P-PRP. Furthermore, compared to P-PRP, L-PRP induced the differentiated NPMSCs to upregulate the expression of TNF-*α* and IL-1*β*, enhanced activation of the NF-*κ*B pathway, increased the expression of MMP-1 and MMP-13, and produced less ECM in differentiated NPMSCs.

**Conclusions:**

Both P-PRP and L-PRP can induce the proliferation and NP-differentiation of NPMSCs. Compared to L-PRP, P-PRP can avoid the activation of the NF-*κ*B pathway, thus reducing the inflammatory and catabolic responses.

## 1. Introduction

As a major cause of low back pain, intervertebral disc degeneration (IDD) is drawing increasing attention for substantial financial and health care burdens worldwide [1]. Although the etiology of IDD is currently unknown, mounting evidence has shown that the mechanical and biological degradation of the discs is considered as one of the common major causes of IDD [2]. Intervertebral disc consists of three distinct structural compositions, the outer annulus fibrosis, the inner NP (nucleus pulposus), and the upper and lower layers of endplates [3]. As the core portion of the intervertebral disc, NP plays a critical role in transmitting the load [4]. The failure of the load transmission is often considered the initiation of the disc degeneration [2]. Thus, the preservation and regeneration of the NP are often the concerns for the therapeutic strategies [5]. Currently, conservative treatments, including oral analgesics and NSAIDs, are clinically applied to alleviate the symptoms [6]. Spinal surgeries, especially those with minimal invasive techniques, can efficiently relieve the symptoms of neural compression. However, the degradation of the intervened or the adjacent discs may undergo an increasing degeneration course [7].

Currently, stem cell transplantation therapy is becoming a promising strategy when transplanted into the degenerated discs [8]. The convincing outcomes were well illustrated in many clinical and basic studies [9–11]. The microenvironment of IVD has the characteristics of low nutrition, acidity, hypertonicity, hypoxia, and high mechanical load [12]. This microenvironment not only has negative influence on original cells of the disc but also promotes apoptosis of transplanted cells [13]. It should be noted that the mesenchymal stem cells (MSCs) reside in the degenerated nucleus pulposus tissues for their regenerative potential. A recent study confirmed that the endogenic MSCs in the nucleus pulposus tissues (NPMSCs) were more resistant to hyperosmotic, acidic, and anoxic environment than the MSCs of fat sources [14]. Thus, in the degenerative disc microenvironment, the activation or transplantation of NPMSCs may have more advantages over MSCs from other tissues. As a useful MSC activator, PRP is widely investigated in tissue engineering strategy for its potential in cell proliferation and extracellular matrix [15–17]. When activated, a variety of growth factors, including PDGF, TGF, EGF, and VEGF, are secreted from the platelets, contributing to a joint regenerative effect on the damaged tissues [18]. Direct injection of PRP has been proven effective in IDD treatment by comprehensive researches [19, 20].

Although PRP is widely used for its regenerative potential, the efficacy was often in debate for its indeterminate therapeutic effect. Some studies revealed that PRP was effective in the repair of tendon injury [21–23], while others did not confirm the functional recovery of the repaired tendon and pain relief of the patients [24–26]. The inconsistency might be caused by the individual difference in patients and different preparations of PRP in each study [27]. Different preparations for PPR bring out various components, and leukocyte is one of the critical elements. Exclusion of leukocytes in PRP has been proven more beneficial for osteoarthritis [28] and bone defects [29]. In addition, PRP rich in leukocytes (L-PRP) can release high concentrations of inflammatory cytokines, which result in the activation of NF-*κ*B pathway [28, 29]. However, the potential harmful effects of leukocytes in PRP on nucleus pulposus-derived mesenchymal stem cells (NPMSCs) have seldom been studied.

The objective of this study is to comparatively evaluate effects of P-PRP and L-PRP on rabbit NPMSCs in vitro, and a new insight is provided to improve the efficiency of PRP treatment in disc regeneration.

## 2. Methods

### 2.1. Preparation of L-PRP, P-PRP, NPMSCs, Spleen Cells, and NPCs

The use of rabbits was approved and supervised by the Animal Care and Use Committee of Southeast University. Autologous whole blood and NPMSCs were harvested from 24 New Zealand white rabbits (8 months old, 3.0–4.0 kg, female) respectively. About 27 ml of autologous whole blood was collected from each New Zealand white rabbit through the carotid artery and mixed with 3 ml acid-citrate dextrose solution A (Santa Cruz, catalog no. SC-214744) to make 30 ml of anticoagulated whole blood. 2 ml whole blood was used in quantifying the platelet and leukocyte concentrations in whole blood, and the rest 28 ml blood was left for preparation of PRP (P-PRP, L-PRP). The method of two-step centrifugation process [30] was applied to prepare the P-PRP and L-PRP. Briefly, 14 ml whole blood was centrifuged at 250g for 10 minutes at room temperature to separate the blood into three layers, platelet-containing plasma at the top, buffy coat (rich in leukocytes and platelets) in the middle, and erythrocytes at the bottom. The top two layers were transferred into a new tube and spun again at 250g for 10 minutes; most of the leukocytes, platelets, and fibrinogen precipitated. Then, most of the supernatant (poor in platelet) was discarded. The left plasma and precipitate, which were almost 2 ml, were resuspended to form the L-PRP. The other 14 ml whole blood was centrifuged at 160g for 10 minutes to separate platelet-containing plasma from buffy coat (rich in leukocytes) and erythrocytes. Then plasma layer was aspirated carefully to avoid the buffy coat and erythrocyte pollution. The plasma layer was centrifuged again at 250g for 15 minutes. Then, most of the supernatant (poor in platelet) was discarded. The left plasma and precipitate, which were almost 1.5 ml, were resuspended to form the P-PRP.

Nucleus pulposus tissue was isolated from IVDs of the New Zealand white rabbits above, minced into 1mm^3^ tissue block, and digested using 0.2 mg/ml type II collagenase (Thermo Fisher, catalog no. DS56580) in DMEM-LG (GIBCO, catalog no. AB10104399) medium for 4–6 h. After centrifugation at 1500 r/min for 10 minutes (DragonLab, D3024R), the cell pellet from the discs (lumbar 3-5) of the same rabbit was cultured in 25 cm^2^ cell culture dish at a density of 10^4^ cells/cm^2^. The cells were cultured in DMEM-LG medium supplemented with 10% fetal bovine serum (FBS) (Sigma, catalog no. BK20170120), 100 U/ml penicillin G (Hyclone, lot: J150019) and 0.1 mg/mL streptomycin (Hyclone, catalog no. K270109) under a humidified atmosphere of 95% air and 5% CO_2_ at 37 °C. The medium was changed every 3 days until the cells reached 80%–90% sub-confluence, then cells were harvested using 0.25% trypsin and 0.02% EDTA (Hyclone, lot: J160004) and re-suspended in the same medium. Then the cell suspension was inoculated into tissue culture dishes at a density of 50 cells/cm^2^ for further culture. The images of cell morphology were performed using microscope attaching camera (OLYMPU, IX51). The NPMSCs of passage 2 were selected for further experiments.

NPCs were isolated and harvested as previously reported [31]. Nucleus pulposus tissue were obtained from the rabbits above and immediately minced into 1 mm3 tissue block and digested using 0.25% trypsin (Thermo Fisher, catalog no. 25200056) for 5 to10 minutes and 0.25% type I collagenase (Thermo Fisher, catalog no. 17100017) for 20 to 25 minutes. After centrifugation (1500 r/min, 10 min), the cell pellets were re-suspended in monolayer culture supplemented with DMEM medium containing 10% fetal bovine serum (Sigma, catalog no. BK20170120), 100 U/ml penicillin G (Hyclone, lot: J150019), and 0.1 mg/ml streptomycin (Hyclone, catalog no. K270109) under standard conditions (37 °C, 21% O_2_, and 5% CO_2_). The medium was changed every 3 days after the primary started to grow by static adherence. The NPCs were collected and subcultured at a ratio of 1:3 until the cells reached 80%–90% subconfluence. The P2 NPCs were used for total RNA extraction.

Spleen cells were isolated according to the method described previously [32]. Briefly, the harvested spleen tissue from the rabbits above was minced and filtrated with 70 *μ*m cell strainer (Corning, catalog no. 431751) to obtain single-cell solution. The cell solution was subsequently centrifuged at 1000 r/min for 10 minutes. The cell pellet was subsequently treated with blood cell lysis buffer (Solarbio, catalog no. R1010-500 ml) to remove red blood cells. The precipitate of mixture of cells was used for total RNA extraction.

### 2.2. Component Analysis of P-PRP, L-PRP, and Whole Blood

The concentrations of leukocyte and platelet in PRP and whole blood were measured by an automatic hematology analyzer (XP-300, Sysmex, Houston, America). The P-PRP and L-PRP were activated with 10% calcium chloride solution and then incubated at 37°C for 7 d. Moreover, the supernatants were extracted from PRP which had been centrifugated at 2800g for 15 minutes. The concentrations of TNF-*α* and IL-1*β* were explored using ELISA kit (Xitang, Shanghai, China) according to the manufacturer's instructions.

### 2.3. Identification of Nucleus Pulposus Mesenchymal Stem Cells

The NPMSCs of passage 2 were subjected to induced differentiation by culturing them in chondrogenic, adipogenic, and osteogenic media, respectively. The cells were evaluated using Alcian blue (Sigma, catalog no. B8438), Oil Red O (Sigma, catalog no. O8010), and alizarin red (Solabio, catalog no. G8550-25), staining respectively. The outcomes were examined by an inverted microscope. RT-PCR was used to determine the expression of MSC (mesenchymal stem cell) mark genes (CD166, CD44, CD29, CD14, CD8, and CD4) from the NPMSCs, NPCs (nucleus pulposus cells), and spleen cells. Briefly, total RNA was extracted from NPMSCs, NPCs, and spleen tissue using TRIzol reagent (Thermo Fisher, catalog no. 10296010) according to the manufacturer's instructions. Reverse transcription was gained by a reverse transcription kit (Thermo Fisher, catalog no.AM334) according to the instruction sequences of the manufacturer. The sequences of primers which were used in the reactions are listed in Table 1.

### 2.4. Proliferation of NPMSCs in Different Concentrations of PRPs (P-PRP, L-PRP)

To determine the cell viability and cell proliferation capacity, cells were examined with CCK-8 assay. The P2 NPMSCs obtained as described above were seeded in a 24-well plate (Yu can Corning/Costar, catalog no. 3415) at 10000 cells per well and maintained in a culture medium containing 2% FBS and P-PRP or L-PRP at various volume percent fractions: 0%, 5%, 10%, 15%, and 20% for 7 days. 100 *μ*l of fresh medium containing 0.5% FBS and 10 *μ*l CCK-8 were added to each plate and incubated for 4 h at 37°C. The optical density was detected at 450 nm, and the experiment was independently performed for three times.

### 2.5. Coculture of NPMSCs In Vitro

The transwell system (Costar, catalog no.JM-3450) was used for coculture of NPMSCs in this study. This transwell consists microporous membrane (0.4um) between upper and lower compartments so that there is free flow of culture medium in the two compartments. The P2 NPMSCs in the basal medium (DMEM + 2% FBS) were seeded into the bottom of the transwell system allocated to three groups (P-PRP, L-PRP, and control). The experimental groups (P-PRP or L-PRP group) were put into 10%P-PRP or 10%L-PRP, respectively, in the top compartment of the transwell system, and the top compartment containing basal medium only was set as the control group. The NPMSCs from all groups were cocultured for 14 days, and the culture medium was changed every 3 days. Concentration of PRPs in this study was adjusted to 10% (vol/vol) using basal medium (DMEM + 2% FBS) according to the proliferation of NPMSC assay above.

### 2.6. Measuring Expression of MMP-1, MMP-13, IL-1*β*, and TNF-*α* in Coculture NPMSCs

Cells from all groups mentioned above were harvested on day 14 by trypsinization and centrifugation. The cell pellet was used to measure cell count by an auto cellometer (Cellometer Auto 2000, Nexcelom, America), and the supernatant was used to estimate the concentrations of MMP-1, MMP-13, TNF-*α*, and IL-1*β* by respective ELISA kits according to the manufacturer's instructions (Xitang, Shanghai, China). Each experiment was repeated in triplicate.

### 2.7. qRT-PCR Analysis

The NPMSCs which were cultured in the transwell described above were harvested on day 14 by 2.5% trypsin and 0.02% EDTA. The gene nucleus pulposus cell-related genes (collagen II and aggrecan), mesenchymal stem cell-related genes (Oct-4, Nanog), inflammatory marker genes (TNF-*α*, IL-1*β*), and catabolic genes (MMP-1, MMP-13) were determined by qRT-PCR. In brief, TRIzol reagent (Invitrogen, CA, USA) was used to extract the total RNA from cells according to the manufacturer's instructions. Real-time PCR was performed using SYBR Premix Ex Taq II (TaKaRa, Dalian, China) and measured on an iQ5 Real-Time PCR Detection System (Bio-Rad, Hercules, CA). The PCR conditions were performed by denaturing the cDNA at 94°C for 4 min, followed by 40 cycles of amplification: 94°C for 40 s, 52°C for 40 s, and 72°C for 40 s for data collection. All samples were normalized to control and calculated using the 2^−ΔΔCT^ analysis method. We used GAPDH expression as the endogenous control, and the sequences of primers which are used in the reactions are listed in Table 1.

### 2.8. Immunofluorescence

The NPMSCs were collected from the experimental groups and control groups as described above. And then, the cells were fixed with PBS containing 4% paraformaldehyde (Sigma, catalog no. D56988) for 20 minutes and washed with PBS including 1% Triton for 5 minutes. Immunostaining for nucleus pulposus cell-related proteins (collagen II and aggrecan) was implemented by blocking the cells in 2% mouse serum (Novus Biologicals, catalog no. NB600-504) and then incubated with mouse anti-rabbit collagen II antibody (Aridobio, catalog no. ARG62450) or anti-rabbit aggrecan (Novus Biologicals, catalog no. NB600-504) at 4°C overnight. The cells were washed 3 times with PBS for 5 minutes each and incubated with Alexa Fluor 594-conjugated goat anti-mouse lgG secondary antibody (Bastet, catalog no. BK0027) for 90 minutes at room temperature. The cell nucleus was counterstained by Hoechst 33342 (Thermo Fisher, catalog no. 62249). The inverted fluorescence microscope (BX53, Olympus, Japan) was used to observe the stained cells.

### 2.9. Western Blot Analysis

The cells from all groups mentioned above were harvested after culturing for two weeks. Extraction of total proteins in the cells was performed by using M-PER (mammalian protein extraction reagent) (Fermenta, catalog no. 26616) supplementing 1.5% (vol/vol) protease inhibitors (Bio-Rad catalog no. 161-0156). Concentration of proteins in the supernatant was measured by using the BCA Protein Assay Kit (Thermo Fisher catalog no. EC60980) according to manufacturer's instruction after centrifugation at 10000 r/min for 10 minutes. 20 *μ*g of total cell protein extracts from each group was separated by 25% SDS polyacrylamide gel electrophoresis (Thermo Fisher, catalog no. DF65896) at 100 V for 60 minutes, then transferred onto PVDF membranes (Millipore catalog no. IPVH00010) at 100 V for 30 minutes which was blocked with 3% fat-free milk in Tris-buffered saline at room temperature for 30 minutes. The blots were incubated with anti-P65 antibody (Abcam, catalog no. ab154036), anti-aggrecan antibody (Novus Biologicals, catalog no. NB600-504), anti-collagen II antibody (Aridobio, catalog no. ARG62450), and anti-GAPDH antibody at a dilution of 1 : 1000 at 4°C overnight, followed by incubation with IgG-HRP (goat anti-mouse peroxidase-conjugated secondary antibody) (Bastet, catalog no. BK0027) at a dilution of 1 : 5000 for 1 h at room temperature. The expression of protein was detected by ECL kit (Biyuntian, catalog no. ce7827) according to the manufacturer's suggested protocols. GAPDH was used as an internal control.

### 2.10. Statistical Analysis

The difference between different two groups of three independent experiments was analyzed by Student's *t*-test. One-way analysis of variance (ANOVA) was used to analyze the difference among more than two groups. The data are presented as means ± S.D. *P* < 0.05 was considered to be statistically significant.

## 3. Results

### 3.1. NPMSCs Possessed the Typical Characteristics of MSCs for Self-Renewing, Clonogenicity, Stem Cell Markers, and Multidifferentiation Potential

**Figure 1:**
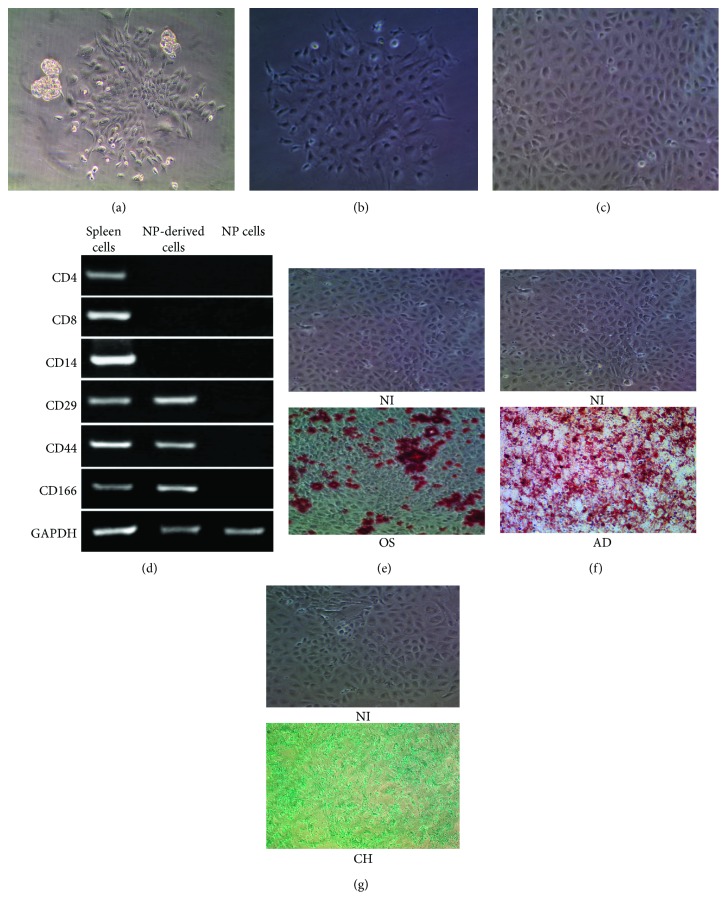
NPMSCs possessed the typical characteristics of MSCs for self-renewing, clonogenicity, stem cell markers, and multidifferentiation potential. (a) The morphology of primary cells (100x). (b) A typical sunflower-like cell colony at passage 1 (100x). (c) Cells displayed a uniform cobblestone-like morphology (100x). (d) The genes of CD29, CD44, and CD166 expressed strongly in cells of passage 2 but not in NP cells and seldom expressed CD14, CD8, and CD4; The spleen cells expressed all genes above as positive controls; the NP cells did not express these genes as a negative control. (e) Micrographs showing accumulation of mineralized calcium deposition in noninduced cells (NI) and osteoinduced cells (OS), as determined by alizarin red staining (100x). (f) Micrographs showing degree of lipid droplets in noninduced cells (NI) and adipoinduced cells (AD) as assessed by Oil red O staining (NI100x, AD400x). (g) Micrographs showing the levels of chondrogenesis in noninduced cells (NI) and chondrogenic induction cell (CH), as measured by Alcian blue staining.

After inoculation, the cells isolated from the nucleus pulposus of the disc started to grow by static adherence after 10–14 days, and the primary cells showed various shapes. They mainly comprise round macrophage-like cells and spindle-shaped fibroblast-like cells (Figure 1(a)). After low-density (50/cm^2^) cell passage, the cells formed typical sunflower-like cell colonies (Figure 1(b)). In addition, the cells displayed a uniform cobblestone-like morphology at passage 2 (Figure 1(c)); RT-PCR was performed to determine the gene expression of typical MSC surface marks. As shown in Figure 1(d), the results indicated that the expression of markers in passage 2 including CD166, CD44, and CD29 which frequently exist in MSCs was significantly higher than that in NPCs. In addition, the markers containing CD14, CD8, and CD4 were seldom expressed which are negative in MSCs. Meanwhile, the cells were induced to differentiation of chondrogenesis, osteogenesis, and adipogenesis respectively (Figures 1(e)–1(g)). When the cells were cultivated in the osteogenic medium, the morphology of the cells changed on the fifth day. The calcium deposits in the cells were highly visible after 3 weeks, and then they were fixed and stained by “alizarin red.” In contrast, the cells in the control group did not produce any calcium deposits which were cultivated in basic medium (Figure 1(e)). After culturing in adipogenic induction medium for 1 week, the cells gradually developed lipid droplets. The cells were stained with “Oil Red O” on day 21. The cells of the control group did not have any change (Figure 1(f)). The cells differentiated into chondrocyte-like cells and emerged a much higher level of “Alcian blue” staining after culturing in a chondrogenic differentiation medium compared with control cells (Figure 1(g)).

### 3.2. The Concentrations of Leukocytes, Inflammatory Cytokines (IL-1*β* and TNF-*α*), and Platelet in Whole Blood, L-PRP, and P-PRP

**Figure 2:**
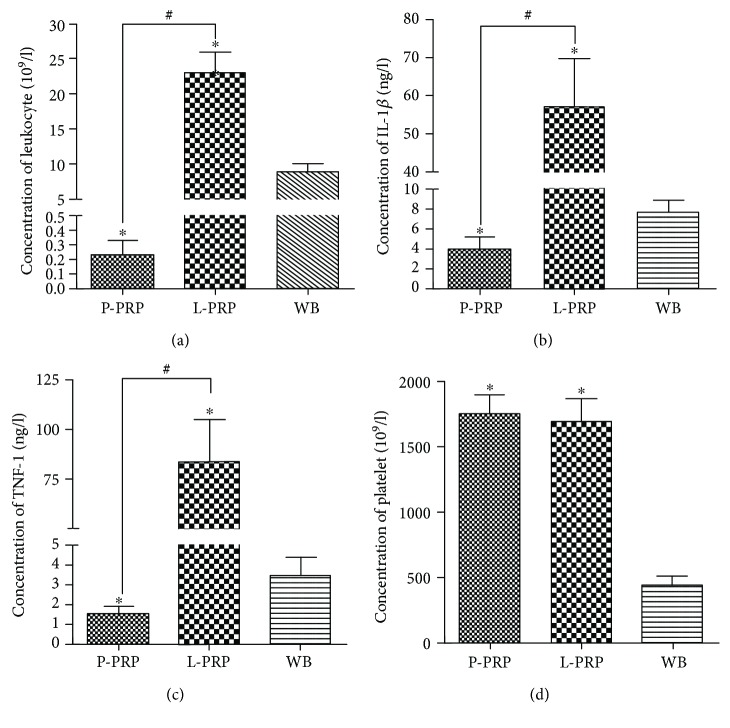
The concentrations of leukocytes, inflammatory cytokines (IL-1*β* and TNF-*α*), and platelet in whole blood, L-PRP and P-PRP. (a–c) The leukocytes, inflammatory cytokines, IL-1*β*, and TNF-*α* in P-PRP, L-PRP, and whole blood. (d) Platelet concentrations of P-PRP, L-PRP, and whole blood. “∗” indicates that the difference between P-PRP or L-PRP and whole blood was statistically significant (*P* < 0.05). “#” indicates that the difference between L-PRP and P-PRP was statistically significant (*P* < 0.05). Statistical analysis using ANOVA, *n* = 8.

We found that the leukocyte concentration in L-PRP was markedly higher than that in whole blood while the concentration of leukocyte in P-PRP was significantly lower than that in whole blood (Figure 2(a), *P* < 0.05). The levels of IL-1*β* and TNF-*α* in L-PRP were elevated. As shown in Figures 2(b) and 2(c), the levels of IL-1*β* and TNF-*α* in L-PRP were significantly higher than those in whole blood and P-PRP, while the levels of IL-1*β* and TNF-*α* in P-PRP were evidently lower than those in whole blood (*P* < 0.05). The concentration of platelet was similar between P-PRP and L-PRP. In addition, concentrations of platelet in P-PRP and L-PRP were 3.8-fold higher than those in whole blood (Figure 2(d)).

### 3.3. The Proliferation of Cells Is Dose-Dependent to PRPs

**Figure 3:**
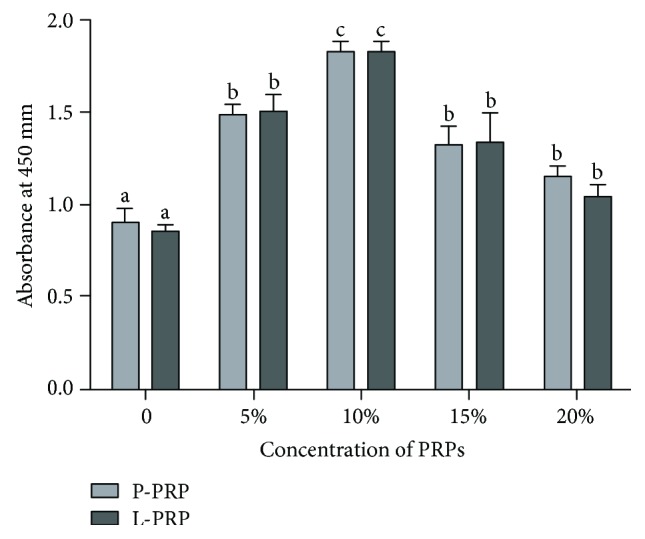
The proliferation of cells derived from nucleus pulposus in different concentrations of P-PRP or L-PRP. Cell proliferation was evaluated on day 7 by CCK-8 assay. The cell proliferation showed a maximum effect on 10% P-PRP and 10% L-PRP. Differences for the date from each group (P-PRP or L-PRP) performed by using one-way analysis of variance. A *t* test was used for determining the statistical significant difference between P-PRP and L-PRP in each concentration of PRPs. Different letters above bars indicate that the difference is statistically significant (*P* < 0.05).

To ascertain whether PRP could functionally regulate proliferation, we performed CCK-8 assays to evaluate the role of PRPs in the progression of cells. When the cells cultured in the medium comprising different concentrations of PRPs as described above, the results displayed that cell proliferation rate showed dose-dependent response on P-PRP and L-PRP (Figure 3). The cell proliferation rate had no significant difference at the presence of 5%, 15%, and 20% P-PRP or L-PRP and increased by 60% compared with the control groups (0% PRPs). The presence of 10% P-PRP or 10% L-PRP obtained the maximum proliferation rate. There was no significant difference between L-PRP and P-PRP in each concentration (*P* > 0.05).

### 3.4. PRPs Promote the Differentiation of NPMSCS into Nucleus Pulposus-like Cells

**Figure 4:**
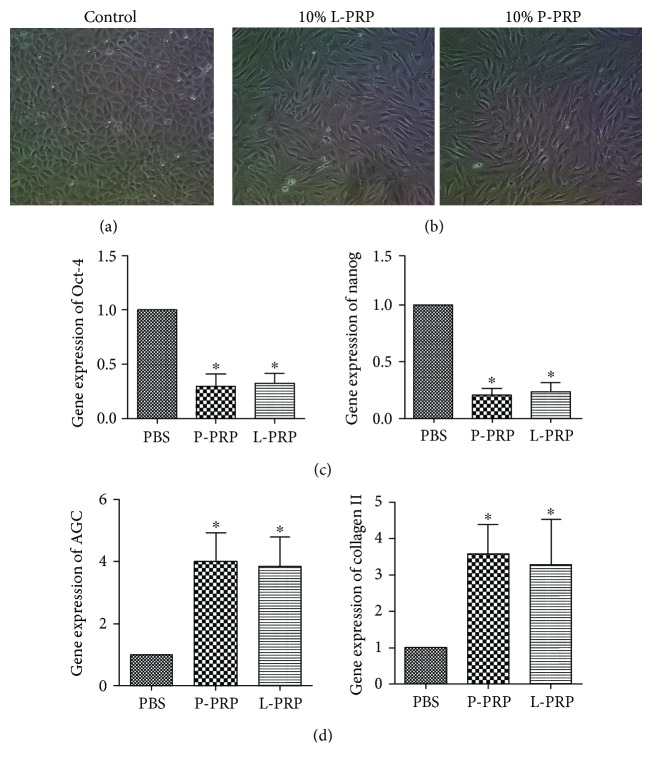
Effects of P-PRP and L-PRP on differentiation in the NPMSCS. (a) Cells morphology in the control. (b) Morphology of the cells treated with 10% L-PRP and 10% P-PRP. (c) mRNA expression of stem cell markers Oct-4 and Nanog, as determined by qRT-PCR. (d) mRNA expression of NP cell-related genes, aggrecan (AGC) and collagen II. “∗” indicates that the difference between P-PRP or L-PRP and control is statistically significant (*P* < 0.05). Note that cell morphology was observed under an inverted microscope (×100).

To observe the effects of P-PRP and L-PRP on differentiation of NPMSCS, we first investigate the cell morphology. The results showed that controls were mainly cobblestone-like without any change (Figure 4(a)), while cell morphology of experimental groups gradually changed from cobblestone-like cells to elongated spindle-shaped nucleus pulposus-like cells (Figure 4(b)). Furthermore, the results of qRT-PCR suggested that the expression of stem cell marker gene including “Oct-4 and Nanog” in the experimental groups decreased 30% compared with that in the control (Figures 4(c)). In contrast, the expression of nucleus pulposus cell-related genes (collagen II and aggrecan) in the experimental groups was 3- to 4-folds higher than that in the control group (Figure 4(d)) Moreover, there was no significant difference between P-PRP groups and L-PRP groups (Figures 4(b)–4(d)). These results indicate that both L-PRP and P-PRP induced the differentiation of NPMSCs towards the mature NP-like cells.

### 3.5. L-PRP Activates NF-*κ*B Pathway in Differentiated Nucleus Pulposus-like Cells

**Figure 5:**
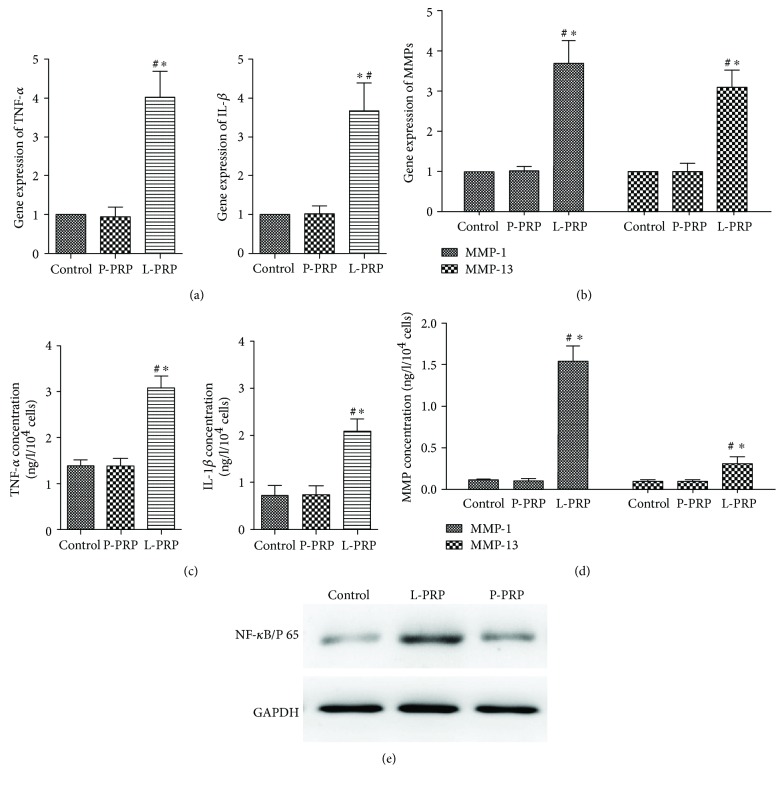
Effects of P-PRP and L-PRP on activation of NF-*κ*B pathway. (a, b) mRNA expression of proinflammatory genes (IL-1*β*, TNF-*α*) and catabolic marker genes (MMP-1, MMP-13), as measured by qRT-PCR. (c, d) Production of proinflammatory cytokines (IL-1*β*, TNF-*α*) and catabolic cytokines (MMP-1, MMP-13), as determined by ELISA assay. (e) Production of NF-*κ*B/p65 in the nucleus, as assessed by western blot. “∗” indicates that the difference between the P-PRP or L-PRP and control was statistically significant (*P* < 0.05). “#” indicates that the difference between L-PRP and P-PRP was statistically significant (*P* < 0.05).

To explore the effects of L-PRP and P-PRP on the activation of NF-*κ*B pathway in the newly nucleus pulposus-like cells, the expression of proinflammatory genes (IL-1*β*, TNF-*α*) and catabolic genes (MMP-1, MMP-13) was determined by qRT-PCR. We observed that the expression of these genes in the L-PRP group was significantly higher than that in the P-PRP and control group (Figures 5(a) and 5(b)). In addition, we used ELISA assay to further investigate these cytokine productions in all groups. The results indicated that the L-PRP group was also significantly higher than P-PRP and control groups (Figures 5(c) and 5(d)). As shown in Figure 5(e), western blot analysis revealed that production of NF-*κ*B/p65 in the L-PRP group was highest in all groups. Moreover, there were no significant differences about all results in the P-PRP and control group.

### 3.6. P-PRP Induces Differentiated Nucleus Pulposus-like Cells to Produce More Extracellular Matrix-Related Proteins

**Figure 6:**
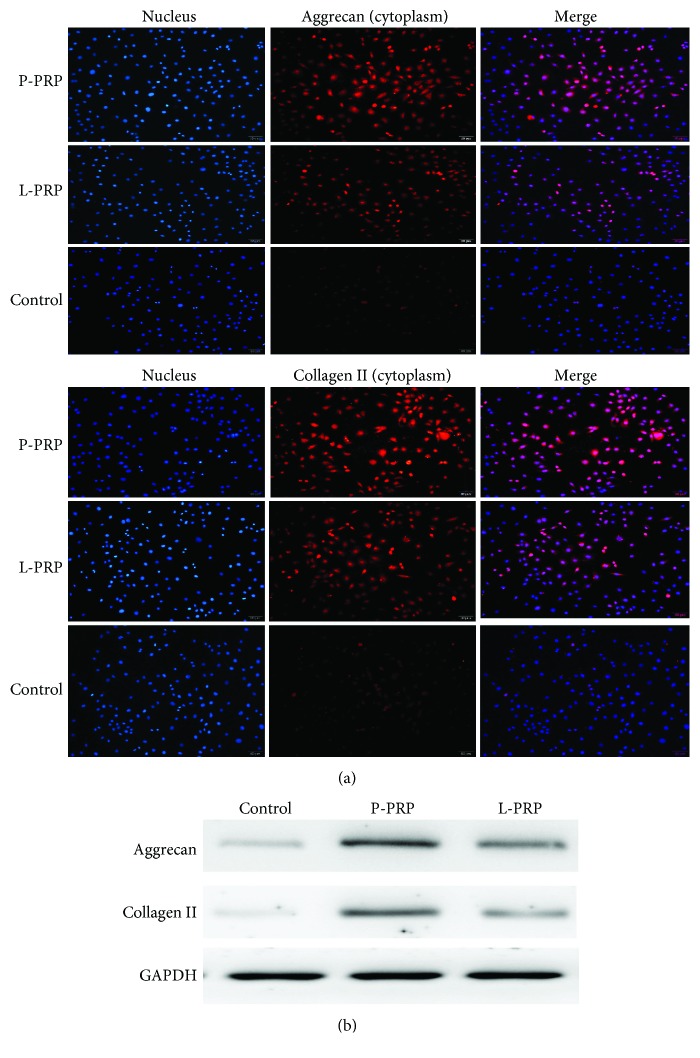
P-PRP induces more extracellular matrix-related proteins. (a) Collagen II and aggrecan in the cytoplasm of the coculture cells imaged by fluorescence microscopy. (b) Production of collagen II and aggrecan, as measured by western blot.

In order to evaluate the effect of P-PRP and L-PRP on productions of extracellular matrix-related proteins in nucleus pulposus-like cells, we measured the levels of collagen II and aggrecan by immunofluorescence staining and western blot analysis, respectively. Immunofluorescence staining showed that PRP treatment upregulated the productions of collagen II and aggrecan when compared with control and the cells obtained the highest staining after P-PRP treatment (Figure 6(a)). We further performed western blot analysis to determine the levels of these factors; the results also identified the result, indicating that P-PRP induced the maximum production of collagen II and aggrecan in cells (Figure 6(b)).

## 4. Discussion

This study determined the significance of leukocyte exclusion in PRP for the culture of NPMSCs in vitro. The isolated NPMSCs possessed the typical characteristics of MSCs for self-renewing, clonogenicity, and multidifferentiation potential. The platelet concentration was over 3 times higher in either P-PRP or L-PRP compared to the whole blood. Concentrations of leukocytes, TNF-*α*, and IL-1*β* were significantly lower in P-PRP compared with those in L-PRP. Both L-PRP and P-PRP induced the differentiation of NPMSCs towards the mature NP cells. P-PRP which induced lower concentrations of MMP-1, MMP-13, TNF-*α*, and IL-1*β* had superior efficacy on the production of ECM (collagen II and aggrecan). In addition, western blot results confirmed the high expression of NF-*κ*B/P65 protein in the L-PRP group.

In the characterization of NPMSCs, we did not test the classic surface markers of MSCs for the lack of specific rabbit antigens by flow cytometry. To solve this problem, we conducted RT-PCT to determine the expression of these markers from the gene level. The colony-forming cells isolated from the NP highly expressed MSC-specific markers (CD29, CD44, and CD166) while minimally expressed the hematopoietic markers (CD8, CD8, and CD14). Spleen cells were used as the positive control for this study, because spleen cells, mainly including lymphocytes and monocytes, are positive for the expression of both MSC-specific and hematopoietic markers [33, 34].

Given the value of endogenic MSCs to the metabolic homeostasis of the disc, an ideal therapy is to activate and proliferate the resident MSC population. PRP, prepared by autologous blood, has been proven effective in the restoration of the degenerated discs [19, 20]. When activated, a variety of growth factors (PDGF, TGF, EGF, and VEGF) released from platelet *α*-granules in PRP are able to increase cell proliferation and cartilaginous matrix secretion in vitro [18]. In this study, both P-PRP and L-PRP had over 3 times higher platelet concentration compared to the whole blood. After 7 days of coculture with PRPs, NPMSCs proliferated faster than the control group. Moreover, NPMSCs exhibited active NPC shape with increased expression of nucleus pulposus cell-related genes (collagen II and aggrecan) and their protein production after treatment of PRPs for 14 days. Therefore, PRPs not only improved cell proliferation but also induced the active differentiation of NPMSCs for upregulating the extracellular matrix-related protein production.

Variations in the composition of the PRP may contribute to distinct results [21]. The exclusion of leukocytes has been proven more effective when applied in the treatment of bone defect [29], osteoarthritis [35], and acute tendon injury [36]. In this study, similar platelet concentrations of P-PRP and L-PRP resulted in significantly different ECM production in each individual group. P-PRP presents better ECM production function, which could be attributed to the exclusion of leukocytes compared to L-PRP. As typical proinflammatory cytokines, IL-1*β* and TNF-*α* are efficient activators of NF-*κ*B signaling pathway [37–39]. In the present study, the proinflammatory genes (TNF-*α* and IL-1*β*) as well as their respective protein expression of differentiated NPMSCs in the L-PRP group were significantly higher than those in the P-PRP group. Western blot results showed the higher expression of NF-*κ*B/P65 protein in the L-PRP group compared with P-PRP. These results are consistent with previous studies showing that the NF-*κ*B pathway is activated by the high concentration of leukocytes in L-PRP [28, 29, 40].

NF-*κ*B signaling pathway is intimately involved in the impaired anabolism and enhanced catabolism by upregulating catabolic cytokines, MMP-1 and MMP-13 in intervertebral disc cells [41]. The anti-inflammatory effect of PRP is well confirmed by previous studies [42, 43]. In addition, PRP cleavage products were reported to be able to terminate the NF-*κ*B pathway and downregulate the production of COX-2 [44]. However, the high concentration of leukocytes in L-PRP may counteract the anti-inflammatory potential of growth factors released from platelets [45]. In our study, L-PRP highly induced the inflammation compared to PRP with negligible leukocytes (P-PRP). In addition, NPMSCs treated with L-PRP upregulated catabolism-related genes (MMP-1, MMP-13) and their proteins compared with P-PRP. Thus, the activation of NF-*κ*B pathway can be largely attributed to the inclusion of leukocytes in PRP. The exclusion of leukocytes from PRP resulted in less production of IL-1*β* and TNF-*α*, thus prohibiting the activation of NF-*κ*B signaling pathway.

Our study has several limitations. First, we preliminarily confirmed the superiority of P-PRP over L-PRP in the extracellular matrix-related protein accumulation of NPMSCs. However, the precise mechanism, especially the activation of NF-*κ*B pathway, should be investigated in depth for further studies. Second, in this study, we only tested the in vitro effects of PRPs on NPMSCs. Cell culture system cannot necessarily guarantee the effect of P-PRP on NPMSCs when injected into animal models. Third, we did not investigate the effect of PRPs on the NPMSCs from the degenerated discs. When applied in a clinic, how PRPs can influence the already degenerated NPMSCs should be determined in further studies.

## 5. Conclusion

We demonstrated that both L-PRP and P-PRP induced the differentiation of NPMSCs towards the mature NP-like cells and exhibited similar proliferation effects on NPMSCs. However, different leukocyte levels contributed to distinct effects on the activation of NF-*κ*B signaling pathway. Concentrated leukocytes in the L-PRP released high levels of proinflammatory cytokines, resulting in the strong activation of NF-*κ*B signaling pathway. Although P-PRP and L-PRP exerted similar proliferation effects on NPMSCs, P-PRP showed superior efficacy on the production of extracellular matrix-related protein. Therefore, when applied in IDD therapy, P-PRP exhibited a superior agent, which could better restore the degenerated ECM accumulation and function by activation of NPMSCs.

## Figures and Tables

**Table 1:** Sequences of primers used for RT-PCR.

Gene	Forward primers (5′-3′)	Reverse primers (5′-3′)
GAPDH	ACTTTGTGAAGCTCATTTCCTGGTA	GTGGTTTGAGGGCTCTTACTCCTT
CD29	GTCACCAACCGTAGCAA	CTCCTCATCTCATTCATCAG
CD44	CGATTTGAATATAACCTGCCGC	CGTGCCCTTCTATGAACCCA
CD166	GGACAGCCCGAAGGAATACGAA	GACACAGGCAGGGAATCACCAA
CD4	GATGGAGGTGGAACTGC	GGAAAGCCCAACACTATG
CD8	GGGTGGAAAAGGAGAAGC	AGGTGAGTGCGGGAGAC
CD14	CAGGTGCCTAAGGGACT	AATAAAGTGGGAAGCGG
IL-1*β*	CGGTCAAGGAGAGGAGCTTAC	GGACTAGCCCTCGCTTATCTTT
TNF-*α*	GGAGAAGCCGGTAGTGGAGAT	GGTCTGGTCACGGTTTGGAA
MMP-1	CGACTCGCTATCTCCAAGTGA	GTTGAACCAGTCTCCGACCA
MMP-13	GGAGGCGAGAACATCAAGCC	CGGCCTTCCCTCGTAGTGA
Oct-4	ACCTTCATCGGAAACTCCAAAG	ACTGTTAGGCTCAGGTGAACT
Nanog	CTGTGGGTTTCTGTGCTGG	CCGGCTTCAAGGCTTTCAG
Collagen II	CAGGATGTCCAGGAGGCT	GCAGTGGCGAGGTCAGTAG
Aggrecan	GGAGCCCGAGCCTATACTATTT	CCCAAGGACCAATCA

## Data Availability

Data used to support the findings of this study are available from the corresponding author upon request.
